# Fathers’ Involvement with Their Children Before and After Separation

**DOI:** 10.1007/s10680-020-09563-z

**Published:** 2020-07-15

**Authors:** Tina Haux, Lucinda Platt

**Affiliations:** 1 School of Social Policy, Sociology, and Social Research, University of Kent, Canterbury, Kent, CT2 7NZ UK; 2 Department of Social Policy, London School of Economics and Political Science, Houghton Street, London, WC2A 2AE UK

**Keywords:** Fathering, Parental separation, Contact, Nonresidential fathers, UK, Millennium Cohort Study

## Abstract

Changes in fathering over the last decades have led to substantially more involvement of fathers in their children’s upbringing. At the same time, high rates of parental separation and subsequent loss of contact fuel concern about separated fathers’ role in their children’s lives. Underlying such concern is the assumption that separation represents a discontinuity in fathers’ parenting. This paper investigates whether fathers’ pre- and post-separation paternal involvement is linked: are fathers with lower levels of contact after separation those who were less involved fathers when co-resident? To answer this question, we draw on a nationally representative UK longitudinal study of children born in 2000–2001 to interrogate the links between fathering before and after separation for 2107 fathers, who separated from their child’s mother before the child was age 11. We show that fathers who were more involved parents prior to separation tend to have more frequent contact after separation, adjusting for other paternal and family characteristics. The size of this association between pre- and post-separation fathering is, however, modest, and even among more involved fathers, intensity of contact declines over time.

**Electronic supplementary material:**

The online version of this article (10.1007/s10680-020-09563-z) contains supplementary material, which is available to authorized users.

## Introduction

In her presidential address to the Population Association of America, Judith Seltzer argued that demographers should focus on family relationships, including those with family members who are not co-resident, and draw out their implications for individuals in both childhood and adulthood (Seltzer 2019). This paper focuses on one such set of relationships: those between fathers and children, and explores contact outcomes following separation. We address a question that has received limited attention in the literature: how far does fathers’ involvement with their young children prior to separation shape the degree and intensity of post-separation contact?

The broader context for our study lies in the changing nature of family forms, including the high proportion of children who now live without both their biological parents at some point during childhood (Thomson 2014), the increase in children born to cohabiting couples (Musick and Michelmore 2015), and the corresponding interest in how families function when not in stable co-residential relationships (McGene and King 2012). An extensive literature has examined both the implications of paternal absence for child outcomes (Adamsons and Johnson 2013) and the contemporary factors associated with paternal involvement in non-intact families (e.g. Cooksey and Craig 1998; Turney and Halpern-Meekin 2017). Yet, despite the research and policy salience of this topic, we know little about how prior circumstances are implicated in different patterns of post-separation contact, and in particular, whether previously more (less) involved fathers maintain higher (lower) contact.

This study is, to our knowledge, the first large-scale nationally representative attempt to address the association between fathering measured before separation and paternal contact reported after separation. We draw on a rich, longitudinal UK data set, charting a representative cohort of children’s and their families’ lives from infancy. This enables us to take a prospective approach to measuring the relationship between fathering before and after separation, by contrast with the small body of existing literature, which has addressed the issue retrospectively (e.g. Kalmijn 2015). We thus avoid the related problems of selection and recall bias. It also means we can explicitly explore the impact of time since separation on contact patterns as these separating families’ lives unfold. We include fathers who were both married to and those cohabiting with the child’s mother prior to separation, enabling us to ascertain if the relationship between fathering and post-separation contact varies with legal marital status. We differentiate forms of pre-separation involvement in line with the wider literature on fathering, and we also distinguish post-separation contact maintenance from intensity of contact. The context of our study is the UK, which has seen relatively high rates of parental separation, particularly among parents of young children (Henz 2016). Our focus on younger children contrasts with much existing literature on post-separation fathering, but captures an important time when contact patterns may become established and are most at risk of dissipating (Kiernan and Mensah 2011).

## Background

### Fathering Before and After Separation

Recent decades have seen an increased role of fathers in child-rearing. Fathers spend more time and are more involved with their children than before (e.g. Bianchi et al. 2006; Gauthier et al. 2004; Lamb 2010; Craig et al. 2014). Despite an extensive body of research exploring aspects of fathering and their consequences for children, there is, nevertheless, little agreement about how to conceptualise or operationalise involved fathering at a time when care remains gendered (Fagan et al. 2014; McMunn et al. 2017). Key parameters of more involved fathering were proposed by Lamb (2010) as engagement, physical accessibility, and responsibility. These dimensions of parenting remain salient despite various refinements (Pleck 2010; Fagan et al. 2014), though the bulk of research has focused on the dimension of engagement and explored paternal behaviours. Studies have also emphasised the relevance of fathers’ emotional availability (e.g. Dermott 2003, 2014). Outside time use studies, a common approach to capture involved fathering is to compile indices of activities, practices, and emotional bonds (Ermisch 2008; Kiernan and Mensah 2011; Norman et al. 2014; Carlson et al. 2017; Hernández-Alava and Popli 2017; Kroll et al. 2016). Such indices may, however, combine activities such as feeding, playing, and reading with emotional bonds, such as perceived closeness, and responsibility for care, which conceptually represent different dimensions of parenting (Fagan et al. 2014). As those authors note, while both quality and quantity are relevant aspects of fathering, frequency of involvement is often more readily captured than quality in large-scale surveys and is arguably less sensitive to cultural specificity. Frequency or multiplicity of activities cannot be assumed to capture the quality of the parent–child relationship, nor the extent to which such activity is independently initiated, but it does imply presence and engagement (Hook and Wolfe 2012).

A qualitatively distinct dimension of fathers’ involvement that has received attention in the literature is ‘sole parenting’ (Wilson and Prior 2010; Hook and Wolfe 2012; Wall 2014). Since many fathering activities take place jointly with or in the presence of the mother (Craig 2006), ascertaining the degree of time spent alone with the child better captures paternal responsibility and confidence (Doucet 2015). Such ‘solo-fathering’ is critical for forming independent bonds ‘unmediated by the presence of the mother’ (Craig 2006, p. 275; Kalmijn 2015; Wilson and Prior 2010; Wall 2014). It also enhances mothers’ trust in fathers’ ability to look after a child by themselves. Looking after his offspring on his own, particularly in the early years, is likely to have implications for a father’s future relationship with the child (Bünning 2015; Norman et al. 2018; Brandth and Kvande 2018). It may therefore be particularly salient for post-separation involvement. Importantly, research on paternal leave suggests that the motivation for solo-fathering is less consequential than the actual experience of taking responsibility for a child without recourse to the mother (Brandth and Kvande 2009, 2018; Bünning 2015).

While fathers in couple families have been taking on a greater role in child-rearing, ‘unprecedented changes’ in the timing, duration, and sequencing of intimate co-residential relationships (Sassler and Lichter 2020, 35; Raley and Sweeney 2020) mean that a substantial proportion of children growing up in the twenty-first century experience parental separation. In the UK, for example, nearly half of all children are not living with both biological parents by the time they reach adulthood (DWP 2013). By contrast with come country contexts, in the UK, levels of non-contact remain stubbornly high: one in ten non-resident parents do not have contact with their children post-separation, and one in four fathers lose touch with their child within 2 years (Lader 2008; Poole et al. 2016).

Given that most non-resident parents are fathers, the literature has explored the potential impacts on children of a reduced role of fathers (Bernardi et al. 2013): how far non-resident fathers remain involved in their children’s lives (e.g. Cheadle et al. 2010), and the consequences for child outcomes (Amato and Gilbreth 1999). Capturing the degree and nature of fathering in post-separation contexts remains, however, challenging. As with the measurement of paternal activity in intact families, frequency of contact is often the primary measure of father involvement (Aquilino 2006; Cheadle et al. 2010). While frequency may only partially represent the nature of the fathers’ involvement, higher frequency is nevertheless likely to reflect a greater propensity to maintain the relationship and to engage in multiple activities. It is also argued to reflect the ways parents think about their parenting behaviour (Baxter 2012). Some forms of activity may, however, represent an intrinsically greater degree of involvement. Overnight stays, particularly for younger children, indicate both parents’ confidence in the ability of the father to carry out caring roles and demonstrate responsibility (Cashmore et al. 2008). They are also associated with greater father–child closeness.

Contact patterns may not, however, fully represent the desired contact patterns of fathers due to the potential of mothers to facilitate or hamper involvement post-separation. This process is often referred to as maternal gatekeeping (Austin et al. 2013). Such gatekeeping is not unidirectional and does not necessarily imply restrictions on paternal access, even when parents’ relationship is poor (Trinder 2008; Haux and Luthra 2019). It is nevertheless important to recognise that observed patterns of contact, particularly for younger children, involve the active facilitation of the parent with care, who may be influenced by considerations that go beyond how good a parent she deems the father to be. Mothers may have greater discretion in meeting fathers’ desire for contact when they were previously cohabiting rather than married to the father, since cohabiting fathers can lack the legal parental status of formerly married fathers.

### The Relationship Between Fathering Before and After Separation

Despite strong policy endorsement for paternal involvement in their children’s lives (Harris-Short 2010; Trinder 2014), evidence on the benefits of post-separation paternal contact remains at best equivocal (Adamsons and Johnson 2013; Amato and Gilbreth 1999; Bernardi et al. 2013; Steinbach 2019; Kalmijn 2015, 2016; Mooney et al. 2009). This raises the question of how far there may be continuity in fathers’ parenting. If more involved fathers are more likely to retain contact, and less engaged fathers are less likely to do so, there would be fewer negative consequences stemming from (less involved) fathers’ absence (cf. Poortman 2018). Similarly, if ‘good’ fathers tend to be both more involved prior to separation and retain a higher intensity of contact afterwards (Goldberg 2015), those children experiencing separation will miss out less.

Studies tracing fathering in couples suggest early involvement of fathers leads to greater involvement throughout childhood (Schober 2012; Norman et al. 2018; Cabrera et al. 2008). Looking at post-separation couples, Westphal et al. (2014) argued that paternal involvement pre-separation and contact post-separation are linked for two reasons. First, involvement pre-separation may convince a family judge to grant more visitation rights and builds trust in the father’s ability to care for the child. Second, paternal investment in the father–child relationship pre-separation tends to generate commitment and a bond on both sides, which is then more likely to survive the separation.

A small corpus of studies has analysed retrospectively reported information on paternal involvement to investigate the connections between fathers’ pre-separation practices, post-separation contact, and their children’s (adult) outcomes. These studies point to the long-term salience of pre-separation behaviours. Kalmijn (2015) drew on a retrospective study of adults to show how paternal involvement in childhood resulted in more positive relationships between fathers and children in adulthood (see also Fortin et al. 2012). Poortman (2018) analysed a study of separated parents who reported retrospectively on pre-separation contact and contemporaneously on contact patterns. She noted that there was a significant (albeit modest) relationship between pre-separation contribution of the father to parenting and subsequent contact. Such retrospective studies, while suggestive, remain susceptible to potential reporting bias. That is, those respondents who have more or better current contact may recall a higher level of paternal involvement. Carlson et al. (2008) using the longitudinal US Fragile Families Study found that positive co-parenting between separated parents was a strong predictor of future contact. However, they focused on families who were not living together when their child was born. Overall, the evidence is partial but suggests that pre-separation parenting practices may contribute to future contact.

### Our Study

We undertake the first large-scale, nationally representative attempt prospectively to address the association between fathering before separation and contact following separation. We investigate the associations between pre-separation fathering and post-separation paternal contact for a nationally representative sample of over 2000 children born in the UK. On the basis of existing literature, we posit that greater pre-separation involvement will be associated with higher levels of post-separation contact. In line with key distinctions in the literature, we study two discrete dimensions of pre-separation fathering: first an index of fathers’ engagement in parenting activities and behaviours, or ‘active fathering’. This measure summarises frequency of different forms of activity undertaken with the child, both care (e.g. feeding) and play or reading. We take it to represent the degree of familiarity and engagement with the child. It covers activities that may be carried out with or without the child’s mother present and which are appropriate to the child’s age. While the extent to which activities included in this measure are carried out by any given father varies, we consider that all represent behaviours that imply the father actively engaging with his child. Second, we examine the frequency with which the father takes on independent responsibility for the child’s care, without the mother present. Such ‘solo-fathering’ represents confidence in care as well as trust between parents. In additional analysis, we also consider emotional bonds as represented by perceived closeness. All measures are reported contemporaneously by the father.

We link these dimensions of pre-separation fathering to post-separation fathering as represented by contact patterns. While an imperfect measure of the quality of time fathers spend with their child post-separation, contact patterns nevertheless provide us with a widely used proxy for ongoing father–child relationships. We distinguish between any versus no contact on the one hand, and contact intensity on the other. We measure contact intensity as both frequency of (any type of) contact, as an indicator of how positive and sustainable the relationship is, and overnight stays, as an indicator of mutual trust and paternal confidence. We expect pre-separation fathering to be related to both contact maintenance and contact intensity, but since contact breakdown represents such a clear disjuncture in father–child relations, we expect measures of intensity to be more sensitive to variations in pre-separation fathering. Moreover, since overnight stays indicate responsibility, confidence and mutual trust, we expect them to be more sensitive to variations in solo-fathering, which are also argued to capture responsibility, confidence, and trust.

Unlike pre-separation involvement, post-separation contact is reported by the mother. We therefore assume that maternal reports are correlated with the father’s actual contact patterns, and that reported contact does not vary systematically with the father’s pre-separation parenting. These are important assumptions but not, we would argue, unrealistic. While, in couples, individuals tend to report larger amounts of housework than their partners attribute to them, the two reports have been shown to be correlated (Gershuny 2000). Similarly, non-resident parents in surveys generally report more frequent contact than resident parents attest (Lader 2008; Mikelson 2008). These reports are, however, based on unmatched mothers and fathers captured in general surveys. Non-resident parents are generally underrepresented in population surveys for a number of reasons only partially related to the child contact (Bryson and McKay 2018). As Bryson and McKay (2018) show, non-resident fathers captured in existing surveys tend to be more involved parents with higher rates of contact than fathers as a whole, potentially accounting for the discrepancy between mothers’ and fathers’ reports of contact. A Norwegian study based on matched pairs found, instead, that differences in reporting were neither large nor systemically distributed (Kitterød and Lyngstad 2014). While we cannot necessarily generalise these findings to the UK, they provide indicative evidence that reports of contact are not systematically biased. For our purposes, the crucial issue is whether, if there *is* any tendency to misreport post-separation contact, it varies systematically with fathers’ prior parenting behaviour. On this, we have no literature to guide us, though we have the advantage compared to other studies that we are not reliant on retrospective reports from mothers (or others).

### Time Since Separation and Child’s Age

Patterns of pre-and post-separation fathering need to be understood within the child’s unfolding life course. Contact patterns are shaped both by the child’s age and by elapsed time since separation. Specifically, contact tends to decline with time since separation, but increases with child age (Cheadle et al. 2010; Cooksey and Craig 1998; Seltzer and Bianchi 1988). This indicates that it is important to adjust for age in order to estimate the impact of time since separation on contact. Given our focus on the early years, we might expect contact patterns to be particularly susceptible to time since separation. We are partly able to disentangle age of child from the time since separation, since, while our data represent a single cohort, separations occur across the period we consider. In line with existing findings, we expect that contact will increase with child’s age at separation, but that, net of child age, contact will decline over time. We anticipate, however, less decline for more involved fathers.

Focusing on time since separation has an additional advantage in helping to address the issue raised by Goldberg (2015), namely whether any association between paternal involvement and post-separation contact is a selection effect. If the relationship between pre- and post-separation paternal involvement is a consequence of the fact that ‘committed’ fathers are both more likely to be involved fathers and to maintain higher levels of contact, even after a separation, we might expect stability over time in contact patterns among more involved fathers. If contact declines even among more involved fathers, then it is less plausible that any observed relationship between pre- and post-separation fathering is purely driven by selection.

### Married or Cohabiting?

As births to cohabiting rather than married couples are on the rise, there is a rich literature charting the extent to which cohabiting couples differ from those who are married (Seltzer 2019; Sassler and Lichter 2020). In the UK, cohabiting couples form a relatively small group of families with dependent children (15% compared to 60% who are married and 25% who are lone parents). However, they are the fastest growing group in the UK (ONS 2015) and are more likely to separate than married couples (Crawford et al. 2012). Previously cohabiting fathers are thus increasingly overrepresented among non-resident parents. The literature, however, is mixed on the extent to which cohabiting fathers’ parenting practices differ from those of married fathers. Some studies find no differences, others find greater involvement from married, and others from cohabiting fathers on specific aspects of parenting (Berger et al. 2018; McClain and DeMaris 2013; Cabrera et al. 2011; Reneflot 2009; Crawford et al. 2012; Manning and Brown 2013).

Turning to post-separation contact, Cheadle et al. (2010) found lower contact among fathers who were not previously married to the child’s mother (see also Seltzer and Bianchi 1988). This has been associated with lower commitment to family life among fathers in cohabitating relationships (Cabrera et al. 2008). However, it might in part also be attributable to fewer rights should the mother choose to restrict access. Despite its increasing prevalence and normalisation (Thomson et al. 2019), there remain social, cultural, and legal differences between cohabitation and marriage (Perelli-Harris and Bernardi 2015). Yet, despite the attention paid to cohabitation in relation to both child outcomes and risks of separation (Crawford et al. 2012; Thomson et al. 2019), we lack clear evidence for the UK on whether paternal involvement plays out differently for married and cohabiting fathers.

Overall, as rates of cohabiting increase, we might expect those who marry to be more highly selected (Thomson et al. 2019) and potentially more committed to family life (Perelli-Harris and Bernardi 2015). On the other hand, the presence of children in cohabiting relationships represents an alternative public form of commitment (Berrington et al. 2015), which might imply fewer differences. Given the enduring legal differences that accompany marriage, cohabiting fathers may face greater barriers in access and additional challenges of staying in touch, negatively impacting levels of post-separation contact. However, we might expect the *relationship* between pre-separation fathering and post-separation contact intensity to be somewhat weaker for cohabiting compared to previously married fathers. Previously married fathers will have greater scope to pursue chosen levels of involvement; while for cohabiting fathers, their levels of post-separation engagement may be more consequential in ensuring ongoing access.

### Additional Factors

Existing research has identified a range of factors associated with contact (Amato et al. 2009; Hunt 2003; Hunt and Roberts 2004; Kalmijn 2015; Lader 2008; Poole et al. 2016; McGene and King 2012). Many such factors are measured post-separation, however, and are affected by the circumstances of the separation and subject to reverse causality. For example, fathers committed to contact will aim to stay more geographically proximate, as well as geographical proximity easing contact. Other factors represent pre-separation circumstances and characteristics that may continue to be relevant, such as parental socio-economic status, health and education, and length of the parental relationship (Lader 2008). We therefore adjust for those pre-separation factors that might confound the relationship between pre-separation father involvement and subsequent contact patterns. We distinguish them in time from the separation itself to avoid reverse causality (cf. Kalmijn 2015). While some studies suggest that fathers are more involved with boys than girls pre-separation (e.g. Poortman 2018), the evidence is not conclusive. Similarly, findings differ as to the gendered nature of post-separation contact (e.g. compare Cheadle et al. 2010 with Grätz 2017). We therefore control for the child’s sex in the main model and provide separate models for boys and girls in the supplementary materials.

## Data and Methods

### Data and Sample

The Millennium Cohort Study (MCS) is a UK-wide cohort study of around 19,000 children born between September 2000 and January 2002 to families resident in the UK. The MCS employed a stratified clustered sampling design to provide a nationally representative sample across the country, with oversampling undertaken to provide analytical power among smaller groups and those more likely to be underrepresented (Plewis 2007).

The original cohort comprised 18,818 children, whose parents were interviewed at home when their child was aged around 9 months. Further surveys have taken place when the cohort children were aged around three, five, seven, 11, 14, and most recently 17 years old. At each survey up to age 14, the main carer (usually the mother) and their co-resident partner (typically the father) were interviewed and completed a short self-completion questionnaire. We use data from the main parent and partner for the first five surveys covering the first 11 years of the children’s lives (University of London 2012a, b, c, d, 2014).^1^

We focus on families where the cohort child was living with two co-resident biological parents at the initial (9 months) survey, and where the parents subsequently separated. We exclude the small number of cases for which: (a) someone other than the mother was the main respondent at the initial or subsequent surveys; (b) the father subsequently died, and (c) the cohort members are twins or triplets, as parenting and partnership dissolution are likely to differ for multiples. This leaves 14,329 MCS children living with both parents at age 9 months. Of these, 2758 were observed to have experienced a separation involving the parents living in different households by or before age 11. Of these, 2107 had valid information on all relevant variables and form our analytic sample. A comparison of core characteristics at baseline across the full 2758 families and the analytical sample shows few differences, though the analytical sample has a slight overrepresentation of married and employed fathers relative to all separating fathers (Supplementary materials, Table S1). We analyse observations relating to these 2107 children across the age 3 to age 11 surveys, constructing a pooled file with 4559 person waves. The sample for overnight stays is slightly smaller, given that overnight stays were only measured from the age 5 survey (2068 children and 4001 person waves).

### Measures

#### Dependent Variables

Our three outcome variables are: contact maintenance, and two measures of contact intensity: contact frequency and overnight stays. *Contact* was asked of the mother at each survey, but at the age 3 survey, the main contact questions refers to contact between the parents (‘Do you have any contact now with [child]’s father?’), while from age 5 onwards, the question referred to contact between the father and child (‘Does [child] have any contact now with [absent parent]?’). In both cases, the subsequent question asking about frequency of contact between father and child is only asked if the mother answers yes to this initial question. We interpret the age 3 contact question as indicating contact (also) with the child, supported by the fact that in very few cases, does the mother answer ‘yes’ to contact and ‘never’ to the following frequency question, and this slight discrepancy is similar for both forms of the contact question. We coded this variable as 1 (any contact) and 0 (no contact). We recoded responses to 0 if the response to the frequency question was ‘never’.

*Frequency of contact* is derived from the question, ‘How often does [absent parent] see [child]?’. Response options were: every day (1); 5–6 times a week (2); 3–4 times a week (3); once or twice a week (4); less often but at least once a month (5); less often than once a month (6); and never (7). We reverse-coded frequency of contact so that a higher value reflects a greater frequency and combined those who no contact with the ‘never’ category.

From the age 5 survey onwards, respondents who answered ‘yes’ to the contact question were asked whether the child *stays overnight* with their absent parent and if so, how often. Response categories are often (1); sometimes (2); rarely (3); and never (4). Again, we reverse-coded, so that a higher value reflects greater frequency, and combined those with no contact with the ‘never’ category.

#### Explanatory Variables

To measure fathers’ engagement in parenting activities and behaviours, which for brevity we term ‘*active fathering*’, we constructed a sum score of frequency of different activities the father may carry out with the child. The MCS carries a detailed range of questions on parenting behaviours asked of both parents.^2^ As a cohort study, the nature of the questions changes with the child’s age. We therefore make use of fathers’ contemporaneous reports of age-appropriate parenting measures. The response options for all the active fathering questions are: more than once a day (1); once a day (2); a few times a week (3); once or twice a week (4); less than once a week (5); and never (6), reverse-coded so that a higher value represents more active fathering. For each pre-separation sweep, we retain the measures that apply to the child at that age. At each age, the measures are correlated, but common measures across surveys/ages are more highly correlated, indicating that there may be some complementarities or specialisation in the specific activities fathers engage in. Our rationale for the measure is that higher frequencies of ‘doing’ any of these things with the child increases familiarity and engagement that may inform subsequent patterns of contact with the child.

At age 9 months, our composite variable comprises: nappy changing, feeding the baby, and getting up in the night; at age three: getting the child ready for bed, and reading to the child; at ages five and seven: the age 3 measures plus telling stories, singing or making music, drawing, going to the park, playing physically active games, and playing with toys. We standardise the scores for each set of age-specific measures so that they are not affected by the number of measures used at the particular age. A higher score represents more active fathering.

For *solo*-*fathering*, how often the father looks after the child on his own was asked at each survey. At all surveys other than age 3, the response categories are the same as for active fathering questions. However, at age 3, they were: never or almost never (1); sometimes (2); usually (3); and always (4). To harmonise responses at age 3 with those at other surveys, we allocated ‘always’ to ‘more than once a day’, ‘usually’ to ‘a few times a week’, ‘sometimes’ to ‘less than once a week’, and ‘never or almost never’ to ‘never’.

A measure of father’s *perceived closeness* to his child covering more emotional bonds was asked only at the age 5 and age 7 surveys. This measure has little variance given c.90% of sample fathers regard themselves as either very or extremely close to their child. We therefore do not therefore include it as one of our main measures of paternal involvement but conduct and report on additional analysis on the reduced (older) sample for whom we have this measure.

We constructed a measure of *time since separation* in months from the relationship history questions asked of the mother at each survey.

We use a measure of *child’s age* in years and fractions of years at the time of separation, centred at the mean age for the pooled sample (6.5 years). Even though each survey is conducted at approximately the same age, there is in fact substantial variation in the children’s ages for any given survey, giving us a nearly continuous distribution of ages across our sample.

Given our interest in potential variation by marital status, we coded whether parents were *married* (0) or *cohabiting* (1), prior to separation.

#### Control Variables

We included controls that the literature indicates might confound any observed relationship between parenting and contact. We measure *relationship duration* in years at the time of the 9-month survey. Given its skewed distribution, we group the measure into eight bands representing 1, 2 3, 4, 5, 6–7, 8–9, or 10+ years. Health is measured as *long*-*term limiting illness* (1, and 0 otherwise). An alternative measure of self-reported general health on an ordinal scale gave the same results. For *father’s education*, we include highest qualification, coded 1 (tertiary); 2 (higher secondary); 3 (lower secondary and other); and 4 (no qualifications, reference category). We include a measure of whether the father was *employed* (0) or not (1) prior to separation. Father’s *age* at the first survey was centred around the mean age of all fathers (age 32). While we hold this age constant, fathers’ age at separation varies, with a mean and median of 34 years.

To capture *family economic circumstances* prior to separation, we include quintile groups of household income. We also include a dummy for *residence in London*, given the limited availability and high costs of housing, which may limit newly separated fathers’ options. Family context and opportunities for paternal involvement are also likely to be shaped by mothers’ work status (Norman et al. 2014), so we include pre-separation *maternal employment* (coded 0 if employed and 1 otherwise). We also control for mothers having *long*-*term limiting illness* (1) or not (0).

We include measures of *older* and *younger biological siblings* to capture the extent to which the father was involved in the family prior to the birth of the cohort child and whether he had subsequent children who might influence contact levels. We explored whether parents’ pre-separation *relationship quality* confounded our results, but as it was not associated with fathering pre- or post-separation, or with marital/cohabitation status, for parsimony, we did not include it in our final models.^3^

Given some evidence that fathers may engage more with boys than girls post-separation (Grätz 2017; Kalmijn 2015, 2016), we included a dummy for the child being a *girl* in pooled models, and in supplementary analysis disaggregated by child sex. In the pooled models we tested interactions between girl and active/solo-fathering. No interactions were statistically significant, so we cannot argue that any apparent differences in fathering in disaggregated models represent significantly different patterns for girls and boys.

All explanatory variables are evaluated prior to separation to avoid issues of reverse causality. For time-varying measures, including our fathering measures, we use that which most closely precedes the separation. Age of the child and time since separation are evaluated at the point the contact outcome is measured.

Descriptives of all variables are given in Table 1 for the total pooled sample and for each survey (outcome) measurement point.

**Table 1:** Descriptive sample statistics of dependent and explanatory variables, pooled analysis sample and by survey sweep, person waves, mean (SD), or percentage. *Source*: MCS, age 9-month, 3, 5, 7, and 11 surveys (University of London 2012a, b, c, d, 2014)

Variable	Full pooled sample (*N* = 4559)^a^	*At*			
		Age 3 survey (*N* = 556)	Age 5 survey (*N* = 1034)	Age 7 survey (*N* = 1246)	Age 11 survey (1723)
*Dependent variables*					
Has contact					
No contact	16.8	17.6	15.2	15.6	18.5
Any contact	83.2	82.4	84.8	84.4	81.5
Frequency of contact (1 = never-6 = every day)	3.48 (1.57)	3.57 (1.65)	3.52 (1.51)	3.48 (1.54)	3.43 (1.60)
Frequency overnight stays (1 = never-4 = often)	2.61 (1.30)	*NA*	2.53 (1.31)	2.70 (1.30)	2.6 (1.30)
*Pre*-*separation fathering*					
Active fathering (standardised, -3.9-2.6)	− 0.04 (1.02)	− 0.03 (0.99)	− 0.08 (1.01)	− 0.02 (1.02)	− 0.04 (1.02)
Solo-fathering (standardised)	0.07 (1.00)	0.01 (0.99)	0.04 (1.01)	0.08 (0.99)	0.10 (0.99)
*Time constant (wave 1 alternatives)*					
Active fathering (standardised)	0.01 (0.99)	− 0.03 (0.99)	− 0.02 (1.00)	0.04 (0.98)	0.03 (0.99)
Solo-fathering	0.03 (1.00)	0.01 (0.99)	0.02 (1.00)	0.03 (1.00)	0.04 (1.00)
*Other measures*					
Months since separation	46.79 (33.18)	16.27 (6.96)	28.90 (15.33)	42.13 (22.48)	70.75 (36.20)
Child age in years (centred around 6.5)	1.13 (2.77)	− 3.31 (0.27)	− 1.28 (0.26)	0.73 (0.26)	4.31 (0.33)
Sex of child					
Boy	51.3	54.1	51.4	50.3	51.0
Girl	48.7	45.9	48.6	49.7	49.0
Marital status					
Married	55.6	41.0	50.0	57.7	62.1
Cohabiting	44.4	59.0	50.0	42.3	37.9
Father’s education					
Tertiary	16.6	13.5	14.1	17.2	18.6
Good secondary	42.9	38.5	43.1	43.7	43.7
Secondary or other	14.2	16.2	13.7	13.8	14.2
None	26.3	31.8	29.0	25.4	23.4
Father’s age at child birth (centred)	− 1.78 (6.43)	− 2.95 (6.57)	− 2.34 (6.35)	− 1.54 (6.47)	− 1.23 (6.33)
Length of parents’ relationship (years)	5.66 (3.79)	4.87 (3.51)	5.43 (3.71)	5.78 (3.84)	5.97 (3.83)
Father’s employment					
Employed	84.8	79.3	82.7	86.0	86.9
Not employed	15.2	20.7	17.3	14.0	13.1
Mother’s employment					
Employed	53.4	45.0	51.2	56.5	60.5
Not employed	44.6	55.0	48.8	43.5	39.5
Father’s health status					
No long-term illness	74.7	73.2	74.7	75.1	74.8
Long-term illness	25.3	26.8	25.3	24.9	25.2
Mother’s health status					
No long-term illness	73.6	72.3	73.4	73.9	73.9
Long-term illness	26.4	27.7	26.6	26.1	26.1
Family income (banded: 1–5)	2.83 (1.28)	2.56 (1.23)	2.70 (1.26)	2.89 (1.28)	2.96 (1.29)
Older natural siblings					
No	54.9	58.3	56.6	54.9	52.9
Yes	45.1	41.7	43.4	45.1	47.1
Younger natural sibling					
No	88.2	100	93.0	87.1	82.2
Yes	11.8	0	7.0	12.9	17.8
Whether family in London					
Not in London	92.8	93.5	93.2	92.8	92.2
In London	7.2	6.5	6.8	7.2	7.8

^a^*N* = 4559 person waves; *N* = 4001 for overnight stays

### Empirical Strategy

We estimate regression models for each of the contact outcomes at each of the surveys at which the child was observed post-separation. For example, where parents separated between the age 3 and age 5 surveys, we use information on post-separation contact at ages 5, 7, and 11. Standard errors are clustered at the child level for all analyses. All estimates account for the complex survey design of the MCS and apply non-response weights.

We estimate binary logistic regressions for any contact and report average marginal effects to render the estimates more comparable with those from the linear regressions (OLS) that we estimate for the intensity measures. As a sensitivity check, we additionally estimated ordered logit models for contact frequency and overnight stays. The results were consistent with those we present here.

For each outcome, we estimate a sequence of five models, two each for each parenting measure, one with just main effects, and one with interactions of fathering with time since separation and cohabitation to test whether more involved fathers were less likely to reduce contact over time, and whether fathering moderated any cohabitation effects. The fifth model includes both of the parenting measures and any significant interactions from the preceding models.

In the logit model of any contact, we can calculate and report marginal effects of fathers’ involvement for those married/cohabiting from models with interactions, but reporting marginal effects for interactions of continuous variables in logistic regressions is not straightforward, and effects cannot be read off from the coefficients as they can with OLS (Ai and Norton 2003). Therefore, for contact maintenance, we established graphically that father’s involvement did not significantly moderate the effect of time across the range of values, and we provide estimates from linear probability models in the supplementary materials (Table S6) showing that the coefficients on time-fathering interactions were small and far from statistical significance.

We present results from models controlling for all covariates, though we report the coefficients only for our key measures of interest. Full results for the final models are provided in the supplementary materials (Table S5). In additional analysis, we re-estimated the main models using constant measures of fathering evaluated at 9 months, with consistent results.

Our strategy for evaluating the role of elapsed time enabled us to adjust for the age of the child concurrently. However, as well as measuring time since separation, the variable could also be capturing an association with age of the child at separation. We therefore estimated additional models to shed further light on the relationship between the age of the child, the time elapsed since separation, and fathers’ contact patterns. We re-estimated all models for each outcome survey sweep (ages 3, 5, 7, and 11) in turn. We also estimated models that explored the coefficient for age of the child within quartiles of the distribution of time since separation (i.e. for those who had experienced separation of 0–19 months, 20–39 months, 40–67 months, and 68 or more months). We discuss the results of these additional models, when we cover the findings on time since separation.

## Results

### Main Results

**Fig. 1:**
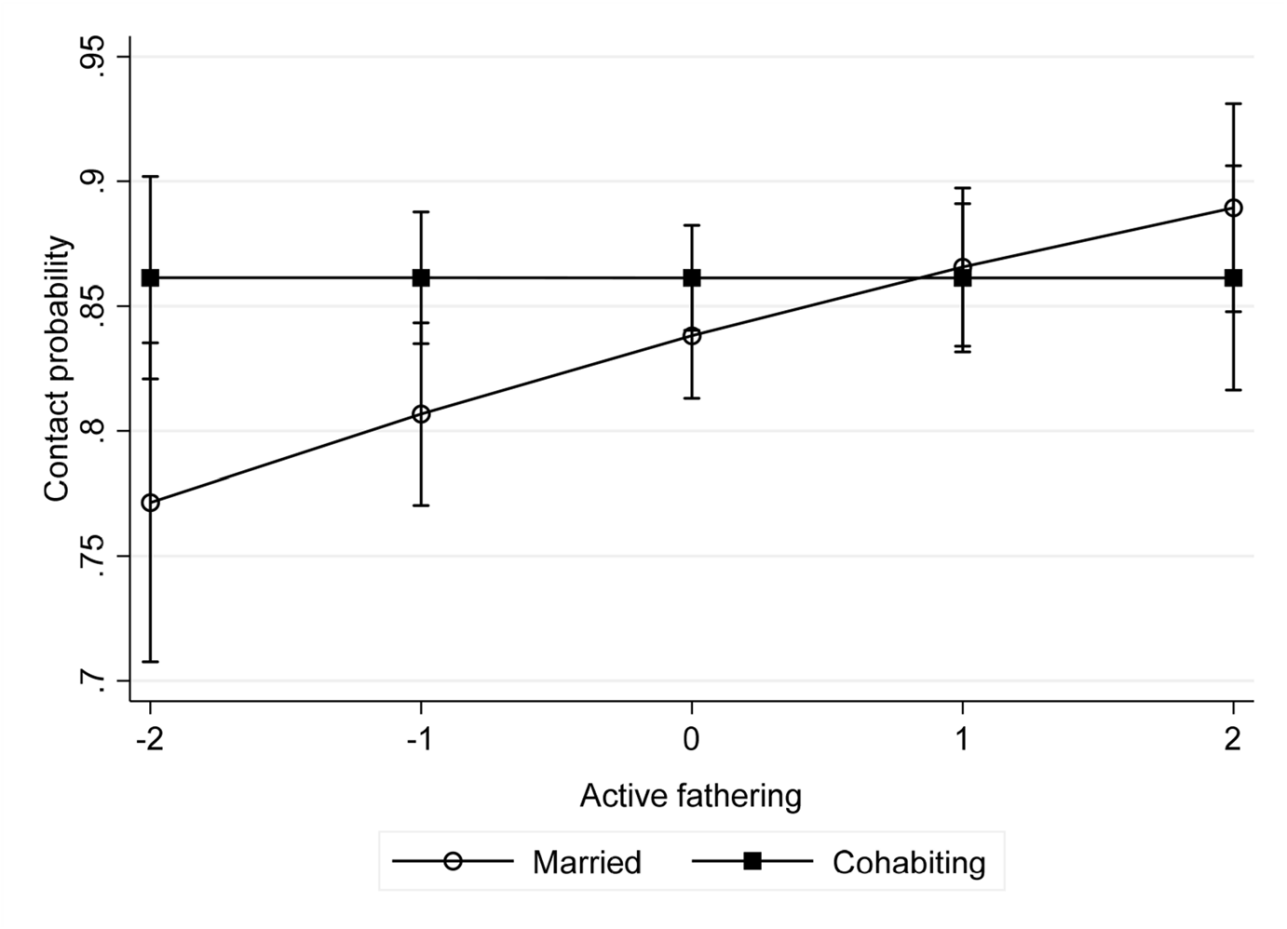
Predictive margins with 95% confidence intervals of contact at different values of active fathering for previously married and previously cohabiting fathers

Tables 2, 3, and 4 report the main results for the three contact outcomes.^4^ Table 2 presents the results for the probability of any contact. It shows that active fathering had a weak net positive association with post-separation contact (Model 1). Model 2 shows that this association is driven by those who were married, since the interaction with cohabitation cancels out the main effect. That is, married men who were more involved prior to separation are more likely to maintain contact, and those who were less involved are less likely to. For those who were cohabiting, contact is independent of their earlier paternal involvement. The interaction between marital status prior to separation and active fathering is illustrated in Fig. 1, showing how cohabiting fathers’ contact is insensitive to pre-separation involvement, while for married fathers, there is a clear, if modest, gradient. Contrary to our expectations, marriage is not associated with higher levels of maintaining contact. Solo-fathering is only marginally significantly associated with maintaining contact; and in the final model, this association drops out altogether, while that for active fathering persists—at least for married fathers. Interacting fathering with time since separation provided no evidence that paternal involvement moderates declines in contact over time (see supplementary materials, Table S6).

**Table 2:** Average marginal effects from logit models of association of fathering involvement with any post-separation contact (*N* = 4559). *Source*: Millennium Cohort Study

	Model 1	Model 2	Model 3	Model 4	Model 5
Active fathering	0.017 (0.008)*	0.026 (0.010)**			0.024 (0.010)*
Solo-fathering			0.015 (0.009)^+^	0.019 (0.010)*	0.011 (0.008)
Months since separation	− 0.002 (0.000)***	− 0.002 (0.000)***	− 0.002 (0.000)***	− 0.002 (0.000)***	− 0.002 (0.000)***
Girl (ref = boy)	− 0.14 (0.016)	− 0.012 (0.016)	− 0.014 (0.016)	− 0.014 (0.016)	− 0.012 (0.016)
Cohabiting	0.027 (0.018)	0.023 (0.018)	0.027 (0.018)	0.026 (0.018)	0.023 (0.018)
Cohabiting * active fathering		− 0.030 (0.015)^+^			− 0.030 (0.015)^+^
Cohabiting * solo-fathering				− 0.018 (0.016)	

All models include additionally: father’s age, qualifications, work status, health status, family income, London or not, mother’s work status and health status, whether child has older or younger siblings, length of the parents’ cohabiting/married relationship prior to the child’s birth. See Supplementary materials for full sets of results. ^+^*p *< 0.1; *p < 0.05; **p < 0.01; ***p < 0.001

**Table 3:** Estimates from OLS models of association of fathering involvement with frequency of contact (*N* = 4556). *Source*: Millennium Cohort Study

	Model 1	Model 2	Model 3	Model 4	Model 5
Active fathering	0.114 (0.035)**	0.128 (0.050)*			0.077 (0.035)*
Solo-fathering	–	–	0.150 (0.036)***	0.205 (0.048)***	0.127 (0.036)***
Months since separation	− 0.013 (0.001)***	− 0.013 (0.001)***	− 0.013 (0.001)***	− 0.013 (0.001)***	− 0.013 (0.001)***
Girl (ref = boy)	− 0.197 (0.067)**	− 0.196 (0.067)**	− 0.195 (0.067)**	− 0.193 (0.067)**	− 0.191 (0.067)**
Cohabiting	0.158 (0.075)*	0.158 (0.075)*	0.170 (0.075)*	0.171 (0.075)*	0.167 (0.075)*
Cohabiting * active fathering	–	− 0.007 (0.070)	–	–	–
Months * active fathering		− 0.000 (0.001)			
Cohabiting * solo-fathering	–		–	− 0.014 (0.073)	–
Months * solo-fathering				− 0.001 (0.000)	
*R*2	0.12	0.12	0.13	0.13	0.13

All models include additionally: father’s age, qualifications, work status, health status, family income, London or not, mother’s work status and health status, whether child has older or younger siblings, length of the parents’ cohabiting/married relationship prior to the child’s birth. See Supplementary materials for full sets of results. *p < 0.05; **p < 0.01 ***p < 0.001

**Table 4:** Estimates from OLS models of association of fathering involvement on frequency of overnight stays (*N* = 4001). *Source*: Millennium Cohort Study

	Model 1	Model 2	Model 3	Model 4	Model 5
Active fathering	0.136 (0.030)***	0.179 (0.049)***			0.113 (0.030)***
Solo-fathering			0.116 (0.031)***	0.186 (0.050)***	0.084 (0.032)**
Months since separation	− 0.004 (0.001)***	− 0.004 (0.001)***	− 0.004 (0.001)***	− 0.004 (0.001)***	− 0.004 (0.001)***
Girl (ref = boy)	− 0.206 (0.058)***	− 0.205 (0.058)***	− 0.209 (0.058)***	− 0.206 (0.058)***	− 0.202 (0.058)***
Cohabiting	0.001 (0.069)	0.001 (0.069)	0.016 (0.069)	0.019 (0.069)	0.008 (0.069)
Cohabiting * active fathering		− 0.005 (0.061)			
Months * active fathering		− 0.001 (0.001)			
Cohabiting * solo-fathering				− 0.088 (0.062)	
Months * solo-fathering				− 0.001 (0.001)	
*R*2	0.14	0.14	0.14	0.14	0.14

All models include additionally: father’s age, qualifications, work status, health status, family income, London or not, mother’s work status and health status, whether child has older or younger siblings, length of the parents’ cohabiting/married relationship prior to the child’s birth. See Supplementary materials for full sets of results. *p < 0.05; **p < 0.01; ***p < 0.001

Turning to frequency of contact (Table 3), we see that both active fathering and solo-fathering are associated with more frequent post-separation contact. In this case, the association does not differ between previously married and previously cohabiting fathers, though cohabiting fathers have higher frequencies of contact. In the final model, when both parenting measures are included, solo-fathering dominates.

**Fig. 2:**
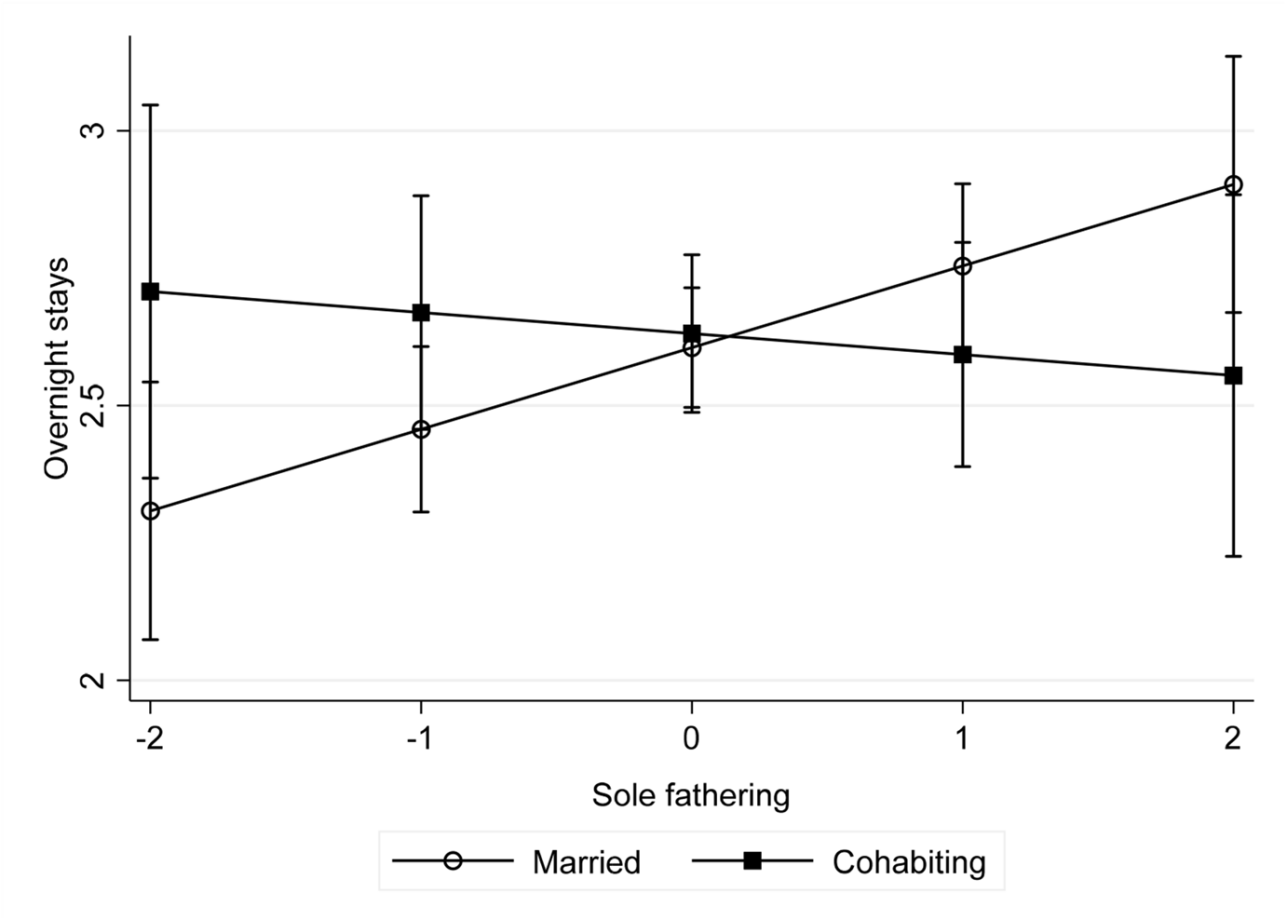
Predictive margins with 95% confidence intervals of regularity of overnight stays at different values of solo-fathering for previously married and previously cohabiting fathers: Sample of girls only

Finally, Table 4 shows that both active- and solo-fathering are separately and independently associated with frequency of overnight stays. These results are replicated for boys. In additional analysis separated by child sex (Table S4), we found that for girls, the association between contact intensity and active fathering is similar for boys, but solo-fathering is only associated with more overnight stays for married fathers of girls (Fig. 2). Time since separation is not moderated by paternal involvement for either measure of contact intensity.

Taken together, these results support the argument that pre-separation fathering is linked to contact post-separation, but that it is more important for contact intensity than for any contact. In fact, for formerly cohabiting fathers, our results provide no evidence of an association between prior paternal involvement and contact maintenance. For contact intensity, both our main measures of fathering involvement seem to play a role. The fact that the picture is rather mixed across the two fathering measures may imply that they both approximate engaged fatherhood, albeit in different ways. Interestingly, both measures of pre-separation fathering show independent associations with overnight stays, which is arguably our most robust measure of contact.

The overall lower probabilities of contact with girls for our measures of contact intensity (see Table S5) are consistent with research that fathers are more likely to engage in joint activities with boys (Poortman 2018). Paternal involvement in the early years may therefore contribute to enhancing fathers’ ongoing relationships with their daughters in particular, should the partnership founder. The fact that there was less evidence for a relationship between paternal involvement and any post-separation contact for fathers who had not been married to the child’s mother may relate to the ways in which maintaining some form of contact becomes more important in the less secure access available to unmarried fathers (Turney and Halpern-Meekin 2017). Thus, specific levels of prior involvement with the child are less salient.

### Time Since Separation

We found no evidence that more involved fathers are more likely to maintain rates of contact over time. Instead for all fathers, the results point to a small but steady decline in contact following separation. For example, each month since separation decreases the chances of having any contact by around 0.2 percentage points. Over a year, this would amount to a reduction of around 2.5 percentage points. These estimates are adjusted for child’s age, which is associated with increasing rates of contact (see Supplementary materials, Table S5): each additional year of age at separation was estimated to increase the chance of contact by around 1.4 percentage points (supplementary materials, Figure S1).

To explore in more detail the role of time since separation and its relationship to the age of the child, we additionally analysed outcomes at individual survey sweeps. We still find a negative association of time since separation at each sweep, while the parenting variables show comparable patterns to the main models, despite the smaller samples (Supplementary materials Table S7). For overnight stays, the measure of time since separation may in part be capturing the impact on contact intensity of splitting up when the child is young, since time since separation is only significantly negative at the most recent sweep. This explanation is intuitive: the longer the father has lived with the child, the more likely he is to maintain more intense contact thereafter.

We also explored how age was associated with contact for discrete durations of time since separation (supplementary materials, Table S8). Child’s age was only significantly associated with greater contact for the longer durations of time since separation, suggesting that when the split has happened more recently, it does not matter whether the child is younger or older, but when there has been substantial time since the break-up, fathers will be more likely to retain contact with relatively older children. Overall, the findings from the different analyses of elapsed time and age at separation suggest that there is fall-off in contact over time, but that older age at separation plays an important part in fathers maintaining greater intensity of contact. These findings are consistent with the literature for the US context (Cheadle et al. 2010; Cooksey and Craig 1998; Seltzer and Bianchi 1988)

### Additional Analysis

We carried out additional analysis on the role of fathering by exploring the measure of closeness captured at the age 5 and age 7 surveys. We found no significant relationship between a father’s perceived closeness to his child and post-separation contact on the reduced sample for which the measure of closeness was available (results not shown). This might be in part due to the lack of variance in the measure, and that parental perceptions of emotional attachment may not in fact reflect more behavioural bonds with the child (Fagan et al. 2014). We also re-estimated the main models using constant measures of fathering reported at age 9 months rather than the time-varying measures. The results were consistent with the ones presented here.

### Other Measures

Results for the other covariates in the model show that fathers’ qualifications and his having been employed prior to separation are positively associated with all forms of contact, while family income was associated with maintaining contact and overnight stays but not contact frequency (Supplementary Materials, Table S5). This is in line with other research that finds family economic context has a bearing on the chances of loss of contact, but not the frequency (Lader 2008). Mothers’ employment is also associated with higher probability of any contact and contact frequency (though not overnight stays), possibly reflecting pre-existing arrangements for dividing care. The length of the parents’ relationship is, as expected, positively associated with greater contact on all three measures. Living in London is associated with both lower frequency contact and fewer overnight stays, consistent with living in a high cost context. Finally, we find no association of fathers’ age at birth, fathers’ or mothers’ health status or presence of biological siblings with contact.

## Discussion

Fathers have become increasingly involved with the upbringing of their children over the past decades, making it relevant to ask to what extent and in what form earlier engagement continues after the end of the romantic relationship. In light of extensive policy and academic interest in post-separation involvement of absent parents in their children’s lives, we aimed to ascertain whether fathers’ post-separation contact patterns showed continuity with their pre-separation fathering involvement. We contribute to the modest literature on this topic by exploiting a nationally representative longitudinal child cohort study to enable us to examine a father’s parenting within an intact family and track his subsequent post-separation contact. We are therefore able to address some of the concerns with response bias in reports of pre-separation fathering in studies reliant on respondent recall. We also address issues of potential reverse causality in cross-sectional studies of determinants of contact by measuring covariates prior to separation.

We anticipated that we would observe links between fathers’ levels of parenting involvement and their post-separation contact patterns, after adjusting for confounders. This was broadly the case, though the size of the association was modest. We also expected—and observed—that contact would decrease with time, a pattern that is a key concern for policy-makers. However, there was no interaction between time since separation and fathers’ involvement: greater early involvement did not slow down contact decline. This suggests that our findings do not purely reflect selection—i.e. that there are not simply certain ‘good’ fathers who are more involved and more likely to retain (more frequent) contact. Similarly, if the association of fathering and contact were primarily driven by mothers facilitating access of formerly more involved fathers, we would not expect contact to decline over time. The fact that there was loss of contact over time regardless of prior paternal involvement demonstrates the challenges in maintaining and supporting a high degree of contact, as lives change and diverge.

We speculated that cohabiting fathers would face greater challenges in maintaining contact, but by the same token, their contact patterns would be more weakly connected to prior fathering behaviour. We found either no difference in levels of contact between married and cohabiting fathers or higher frequency among cohabiting fathers. This finding may align with the ways in which having children is itself a form of public commitment facilitating contact (Berrington et al. 2015). At the same time, we did find the anticipated weaker relationship between fathering and any contact for unmarried fathers. This weaker association was also observed for overnight stays for unmarried fathers of girls—perhaps consistent with the greater gender egalitarianism posited for cohabiting relationships (McClain and Demaris 2013). Given the increasing numbers of children experiencing parental separation of formerly unmarried parents, these preliminary findings on how contact patterns differ by marital status merit further investigation.

Our study has its limitations. Our sample is necessarily selected by including only those families that continue in the study up to and after separation. We have nothing to guide us on how such maintenance within the sample might be related to any association between fathering and (reported) contact. By conditioning on separation our sample may also have specific unobserved characteristics related to their family commitment, which also have a bearing on fatherhood. We have adjusted for a wide range of individual and family factors to reduce the potential influence of unobserved characteristics, but we cannot dismiss the possibility that they may play a role. In common with the majority of large-scale quantitative studies, we are dependent on measures that are defined primarily in terms of quantity of involvement and contact rather than quality. We attempted to address this issue in part by using measures that capture paternal responsibility as well as those simply covering frequency of activity, given frequencies are likely to encompass heterogeneous quality of fathering.

Despite these limitations, we have demonstrated that paying attention to pre-separation circumstances has the potential to pay dividends when aiming to understand patterns of post-separation contact. Contact has an association with fathers’ behaviour prior to separation, indicating that supporting the involvement of fathers in parenting early in the child’s life and through their early years may have payoffs in terms of maintenance and intensity of subsequent contact.

Our findings also indicate that it is important to consider how realistic expectations of more equal sharing of parenting post-separation are, unless there are commensurate increases in—and support for—fathers’ early participation in child care in the family home (Harris-Short 2010). Therefore, policies that encourage and enable fathers to be actively involved with their children, including taking sole responsibility for their children, may play a small, but nevertheless significant, role when it comes to contact arrangements post-separation. Furthermore, enabling fathers to be more involved with their children in intact and separated families would meet the wishes of many fathers and mothers alike. The UK is lagging behind many of its European neighbours when it comes to the provision and uptake of parental leave and achieving a better work-life balance that would allow both parents to spend more time with their children. The goal of creating a better work-life balance for parents in the UK enjoys broad support, and our paper has demonstrated that it is likely to have positive influence on children staying in contact with both parents should they separate.

## Electronic supplementary material

Below is the link to the electronic supplementary material.

Supplementary material 1 (PDF 426 kb)

## Footnotes

1 Our interest is in early childhood (pre-teens), hence our focus on the first five surveys. Moreover, at age 11, the parenting questions were abbreviated and do not provide the same potential for exploring the relationship with subsequent contact patterns, while at age 14, the measurement of contact changed.

2 As the study asks the parents about their own parenting separately, we have no maternal report of fathering that would enable us to cross-check the consistency of their reports.

3 We also checked that post-separation relationship quality was not confounding our results. While we might expect post-separation relationship quality and contact to be endogenous, for clarity, we checked that mothers’ report of post-separation relationship quality was not mediating any association between pre-separation fathering and post-separation contact. Relationship quality was not collected at every wave, but for the reduced sample for which we had the measure, we found no evidence that post-separation relationship quality was mediating the association between pre-separation fathering and post-separation contact—i.e. coefficients for the fathering measures remained very similar in models with and without it.

4 Separate models for boys and girls are provided in the supplementary materials (Tables S2–S4); however, even though levels of contact differed by sex (supplementary materials, Table S5), interactions between child sex and fathering in pooled models were not significant, leading us to focus on the results from the pooled model. The exception was the three-way interaction between child sex, fathering, and married/cohabiting, which we discuss in the text.
