# Supplementary material for: Fathers’ Involvement with Their Children Before and After Separation
Source: Eur J Popul. 2020 Jul 15;37(1):151–77. doi: 10.1007/s10680-020-09563-z (PMC7865041; doi:10.1007/s10680-020-09563-z)
Supplement: Supplementary file 1 — Supplementary material 1 (PDF 426 kb) [file 10680_2020_9563_MOESM1_ESM.pdf]

Fathers' involvement: Supplementary online materials

Table S1: Comparison of sweep 1 characteristics in responding families in reduced analysis samples compared to all families experiencing separation, and all person waves, with characteristics measured pre-separation

	All separating families: N=2758 families	Analysis sample for contact: N=2107 families	Analysis sample for overnight stays: N=2068 families
Sex of child			
Boy	51.7	51.5	51.4
Girl	48.3	48.5	48.6
Marital status			
Married	50.4	53.7	54.0
Cohabiting	49.6	46.3	46.0
Father's education*			
Tertiary	17.3	17.7	17.9
Good secondary	41.9	42.9	42.9
Lower secondary or other	14.2	14.1	14.1
None	26.6	25.3	25.1
Father's employment			
Employed	81.7	84.3	84.5
Not employed	18.3	15.7	15.5
Father's age centred	-1.7	-1.6	-1.5
Income* (banded):1-5	2.7	2.8	2.8
Older natural siblings			
No	54.4	54.2	54.0
Yes	45.6	45.8	46.0
Length of parents' relationship (years)*	5.9	5.8	5.9
Mother's employment			
Employed	52.0	51.6	51.7
Not employed	48.0	48.4	48.3
Father's health status*			
No long-term illness	76.2	76.4	76.5
Long-term illness	23.8	23.6	23.5
Mother's health status			
No long-term illness	74.5	74.2	74.4
Long-term illness	25.5	25.8	25.6
Family lives in London			
Not in London	90.4	92.5	92.5
In London	9.6	7.5	7.5

Notes: Unweighted percentages, apart from length of relationship: mean (SD). Column 1 is based on full response for all 2758 families except for those marked *, where response Ns were as follows: father's education: N=2258; father's health status N=2321; income band: N=2750; mother's health status: N=2756; length of parents' relationship 2576

Table S2. Average marginal effects from logit models of association of fathering involvement with any post-separation contact, separate models for boys and girls

			Boys (N=2338)		
	Model 1	Model 2	Model 3	Model 4	Model 5
**Active fathering**	0.023<br>(0.011)*	0.019<br>(.028)			0.017<br>(0.010)
**Solo-fathering**			0.024<br>(0.012)*	0.017<br>(0.013)	0.019<br>(0.011)+
Months since separation	-0.002<br>(0.000)***	-0.002<br>(0.000)***	-0.002<br>(0.000)***	-0.002<br>(0.000)***	-0.002<br>(0.000)***
Cohabiting	0.016<br>(0.022)	0.016<br>(0.022)	0.017<br>(0.022)	0.017<br>(0.022)	0.018<br>(0.022)
*Cohabiting * active fathering*		-0.001<br>(.021)			
*Cohabiting * solo-fathering*				0.006<br>(0.021)	
			**Girls (N=2221)**		
	Model 1	Model 2	Model 3	Model 4	Model 5
**Active fathering**	0.013<br>(0.011)	0.036<br>(0.015)*			0.033<br>(0.015)*
**Solo-fathering**			0.002<br>(0.012)	0.018<br>(0.014)	0.009<br>(0.013)
Months since separation	-0.002<br>(0.001)***	-0.002<br>(0.001)***	-0.002<br>(0.001)***	-0.002<br>(0.001)***	-0.002<br>(0.001)***
Cohabiting	0.035<br>(0.027)	0.025<br>(0.027)	0.037<br>(0.027)	0.33<br>(0.027)	0.024<br>(0.027)
*Cohabiting * active fathering*		-0.055<br>(0.021)**			-0.047<br>(0.021)*
*Cohabiting * solo-fathering*				-0.042<br>(0.023)+	-0.029<br>(0.023)

Source: Millennium Cohort Study. Notes: All models include additionally: father's age, qualifications, work status, health status, family income, London or not, mother's work status and health status, whether child has older or younger siblings, length of the parents' cohabiting / married relationship prior to the child's birth. See Supplementary materials for full sets of results. + *p*<0.1; * *p*<0.05; ** *p*<0.01; ***p<0.001

Table S3. Estimates from OLS models of association of fathering involvement with frequency of contact, separate models for boys and girls

			**Boys (N=2336)**		
**Active fathering**	0.103 (0.051)*	0.067 (0.072)			0.058 (0.051)
**Solo-fathering**	--	--	0.166 (0.053)**	0.204 (0.070)**	0.148 (0.054)**
Months since separation	-0.013 (0.002)***	-0.013 (0.002)***	-0.013 (0.002)***	-0.013 (0.002)***	-0.013 (0.002)***
Cohabiting	0.142 (0.103)	0.141 (0.103)	0.154 (0.104)	0.151 (0.103)	0.157 (0.104)
*Cohabiting * active fathering*	--	0.102 (0.103)	--	--	--
*Months * active fathering*	--	-0.000 (0.001)	--	--	--
*Cohabiting * solo-fathering*	--	--	--	0.031 (0.105)	--
*Months * solo-fathering*	--	--	--	-0.000 (0.001)	--
**R2**	0.11	0.11	0.12	0.12	0.12
			**Girls (N=2220)**		
**Active fathering**	0.123 (0.048)*	0.182 (0.071)*			0.097 (0.048)*
**Solo-fathering**	--	--	0.122 (0.047)**	0.205 (0.066)**	0.094 (0.047)*
Months since separation	-0.012 (0.002)***	-0.012 (0.002)***	-0.012 (0.002)***	-0.012 (0.002)***	-0.012 (0.002)***
Cohabiting	0.187 (0.106)+	0.181 (0.106)+	0.201 (0.106)+	0.201 (0.106)+	0.188 (0.106)+
*Cohabiting * active fathering*	--	-0.113 (0.092)			--
*Months * active fathering*		-0.000 (0.001)			
*Cohabiting * solo-fathering*	--	--	--	-0.085 (0.102)	--
*Months * solo-*				-0.001 (0.001)	

fathering					
**R2**	0.10	0.10	0.10	0.10	0.10

Source: Millennium Cohort Study. Notes: All models include additionally: father's age, qualifications, work status, health status, family income, London or not, mother's work status and health status, whether child has older or younger siblings, length of the parents' cohabiting / married relationship prior to the child's birth. See Supplementary materials for full sets of results. + p<0.1; * p<0.05; ** p<0.01 ***p<0.001

Table S4. Estimates from OLS models of association of fathering involvement on frequency of overnight stays, separate models for boys and girls

	Model 1	Model 2	Model 3	Model 4	Model 5
**Boys (N=2177)**					
**Active fathering**	0.122 (0.042)**	0.094 (0.070)			0.101 (0.042)*
**Solo-fathering**			0.102 (0.045)*	0.126 (0.069)+	0.071 (0.045)
Months since separation	-0.003 (0.001)*	-0.003 (0.001)*	-0.003 (0.001)*	-0.003 (0.001)*	-0.003 (0.001)*
Cohabiting	-0.025 (0.094)	-0.026 (0.093)	-0.021 (0.095)	-0.020 (0.095)	-0.017 (0.094)
*Cohabiting * active fathering*	--	0.085 (0.086)	--		--
*Months * active fathering*		0.000 (0.001)			
*Cohabiting * solo-fathering*	--	--	--	-0.008 (0.085)	--
*Months * solo-fathering*				0.000 (0.001)	
R2	0.13	0.14	0.13	0.13	0.14
**Girls (N=1965)**					
	Model 1	Model 2	Model 3	Model 4	Model 5
**Active fathering**	0.162 (0.043)***	0.265 (0.066)***			0.143 (0.043)**
**Solo-fathering**			0.126 (0.043)**	0.226 (0.072)**	0.149 (0.053)**
Months since separation	-0.004 (0.001)**	-0.004 (0.001)**	-0.004 (0.001)**	-0.004 (0.001)*	-0.004 (0.001)**
Cohabiting	0.028 (0.098)	0.022 (0.098)	0.053 (0.098)	0.053 (0.098)	0.026 (0.098)
*Cohabiting * active fathering*		-0.086 (0.085)			
*Months * active fathering*		-0.001 (0.001)			
*Cohabiting * solo-fathering*				-0.163 (0.091)+	-0.187 (0.091)*

*Months * solo-fathering*				-0.001<br>(0.001)	
**R2**	0.15	0.15	0.14	0.15	0.16

Source: Millennium Cohort Study. Notes: All models include additionally: father's age, qualifications, work status, health status, family income, London or not, mother's work status and health status, whether child has older or younger siblings, length of the parents' cohabiting / married relationship prior to the child's birth. See Supplementary materials for full sets of results. + p<0.1; * p<0.05; ** p<0.01; ***p<0.001

Table S5: Full model results for logistic regression models of any contact, and OLS models of contact frequency and overnight stays result from full model (model 5)

	Any contact (AMEs)	Contact freq. (OLS)	Overnight stays (OLS)
Active fathering	0.024	0.077	0.113
	(0.010)*	(0.035)*	(0.030)***
Solo-fathering	0.011	0.127	0.084
	(0.008)	(0.036)***	(0.032)**
Cohabiting	0.027	0.167	0.008
	(0.018)	(0.075)*	(0.069)
Cohabiting*active fathering	-0.030	--	--
	(0.015)+		
Time since separation	-0.002	-0.013	-0.004
	(0.000)***	(0.001)***	(0.001)***
Age of child (centred)	0.012	0.064	0.015
	(0.004)**	(0.015)***	(0.013)
Girl	-0.012	-0.191	-0.202
	(0.016)	(0.067)**	(0.058)***
Father's education (ref=none)			
Tertiary	0.097	0.197	0.496
	(0.026)***	(0.116)+	(0.098)***
Good secondary	0.049	0.090	0.192
	(0.022)*	(0.097)	(0.082)*
Lower secondary or other	0.008	0.079	0.055
	(0.027)	(0.125)	(0.103)
Father not employed prior to separation	-0.099	-0.490	-0.532
	(0.023)***	(0.127)***	(0.097)***
Father's age (centred)	-0.000	-0.001	0.000
	(0.001)	(0.006)	(0.005)
Family income prior to separation	0.018	0.036	0.109
	(0.009)+	(0.035)	(0.031)***
Older natural siblings	0.022	-0.042	-0.024
	(0.020)	(0.089)	(0.078)
Younger natural siblings	0.022	0.040	-0.039
	(0.033)	(0.116)	(0.102)
London	-0.000	-0.253	-0.419
	(0.033)	(0.116)*	(0.110)***
Mother not employed prior to separation	-0.042	-0.185	-0.097
	(0.018)*	(0.078)*	(0.071)
Father had limiting health condition	0.013	0.069	0.027
	(0.018)	(0.078)	(0.066)
Mother had limiting health condition	-0.009	-0.092	-0.108
	(0.016)	(0.076)	(0.063)+
Length of parents' relationship	0.012	0.074	0.044
	(0.005)*	(0.020)***	(0.017)*
R2		0.13	0.14

N	4559	4556	4011

+ p<0.1; * p<0.05; ** p<0.01; *** p<0.001

Table S6: Linear probability models of any post-separation contact including interaction between fathering involvement and time since separation

	With active fathering	With solo-fathering
Active fathering	0.032	
	(0.011)**	
Solo-fathering		0.022
		(0.011)*
Cohabiting	0.025	0.027
	(0.018)	(0.018)
Cohabiting*active fathering	-0.024	
	(0.018)	
Cohabiting*solo-fathering		-0.015
		(0.019)
Months since separation	-0.002	-0.002
	(0.000)***	(0.000)***
Active fathering*time since separation	-0.000	
	(0.000)	
Solo-fathering*time since separation		-0.000
		(0.000)
Age of child (centred)	0.008	0.007
	(0.003)*	(0.003)*
Girl	-0.012	-0.014
	(0.016)	(0.016)
Paternal qualifications (ref.=none)		
Tertiary	0.089	0.095
	(0.027)**	(0.027)***
Good GCSEs	0.058	0.062
	(0.025)*	(0.024)*
Low GCSEs	0.016	0.016
	(0.031)	(0.031)
Father unemployed prior to separation	-0.157	-0.156
	(0.034)***	(0.034)***
Father’s age (centred)	-0.000	-0.000
	(0.002)	(0.002)
Family income pre-separation	0.016	0.018
	(0.008)+	(0.008)*
Older (natural) siblings	0.029	0.028
	(0.020)	(0.020)
Younger (natural) siblings	0.030	0.034
	(0.026)	(0.026)
London	-0.006	-0.006
	(0.032)	(0.033)
Mother not in work prior to separation	-0.052	-0.048
	(0.018)**	(0.018)**
Father had limiting health condition	0.017	0.014

	(0.019)	(0.019)
Mother had limiting health condition	-0.008	-0.007
	(0.018)	(0.018)
Length of parents' relationship (years)	0.011	0.011
	(0.005)*	(0.005)*
R^2	0.12	0.12
N	4,559	4,559

+ *p*<0.1; * *p*<0.05; ** *p*<0.01; *** *p*<0.001

Table S7: Additional analysis of time since separation: analysis by survey sweep of separation

	Separated by Age 3 survey	Separated by Age 5 survey	Separated by Age 7 survey	Separated by Age 11 survey
	**Any contact**			
Active fathering	0.031	0.014	0.009	0.014
	(0.016)*	(0.010)	(0.010)	(0.010)
Solo-fathering	-0.006	0.017	0.009	0.014
	(0.017)	(0.012)	(0.011)	(0.010)
Cohabiting	0.074	-0.016	0.029	0.036
	(0.037)*	(0.025)	(0.024)	(0.021)+
Months since	-0.007	-0.003	-0.002	-0.002
separation	(0.002)**	(0.001)***	(0.001)***	(0.000)***
Girl	0.009	-0.039	-0.022	0.008
	(0.035)	(0.022)+	(0.020)	(0.019)
N	556	1,034	1,246	1,723
	**Frequency of contact**			
Active fathering	0.125	0.127	0.042	0.050
	(0.075)+	(0.050)*	(0.045)	(0.044)
Solo-fathering	0.097	0.144	0.081	0.157
	(0.079)	(0.050)**	(0.047)+	(0.043)***
Cohabiting	0.165	0.229	0.179	0.144
	(0.154)	(0.108)*	(0.098)+	(0.091)
Months since	-0.044	-0.021	-0.017	-0.011
separation	(0.012)***	(0.003)***	(0.002)***	(0.001)***
Girl	0.172	-0.293	-0.283	-0.151
	(0.145)	(0.096)**	(0.086)**	(0.078)+
R2	0.14	0.16	0.16	0.13
N	556	1,034	1,243	1,723
		**Overnight stays**		
Active fathering		0.138	0.112	0.097
		(0.047)**	(0.042)**	(0.037)**
Solo-fathering		0.078	0.037	0.120
		(0.046)+	(0.043)	(0.038)**
Cohabiting		-0.015	0.035	-0.001
		(0.105)	(0.094)	(0.078)
Months since		-0.004	-0.003	-0.004
separation		(0.003)	(0.002)	(0.001)***
Girl		-0.265	-0.191	-0.161
		(0.087)**	(0.077)*	(0.068)*
R2		0.16	0.16	0.14
N		1,034	1,244	1,723

Source: Millennium Cohort Study. All models include additionally: child sex, father's age, qualifications, work status, health status, family income, London or not, mother's work status and health status, whether

child has older or younger siblings, length of the parents' cohabiting / married relationship prior to the child's birth. + $p<0.1$; * $p<0.05$; ** $p<0.01$; *** $p<0.001$

Table S8: Additional analysis of child age by duration of separation

	Separated 0-20 months	Separated 21-40 months	Separated 41-68 months	Separated 69+ months
		**Any contact**		
Active fathering	0.498	0.132	0.184	0.279
	(0.158)**	(0.138)	(0.156)	(0.135)*
Solo-fathering	0.139	0.119	0.075	0.071
	(0.126)	(0.117)	(0.117)	(0.100)
Cohabiting	0.210	0.089	0.337	0.202
	(0.284)	(0.248)	(0.239)	(0.225)
Child age	0.070	0.095	0.123	0.165
	(0.068)	(0.059)	(0.060)*	(0.085)+
Girl	0.038	-0.176	-0.195	-0.066
	(0.253)	(0.225)	(0.210)	(0.182)
N	1,180	1,117	1,172	1,090
		**Frequency of contact**		
Active fathering	0.119	0.055	-0.000	0.137
	(0.050)*	(0.053)	(0.048)	(0.057)*
Solo-fathering	0.139	0.199	0.103	0.082
	(0.050)**	(0.051)***	(0.055)+	(0.054)
Cohabiting	0.125	0.134	0.295	0.153
	(0.103)	(0.108)	(0.111)**	(0.123)
Child age	0.027	0.079	0.085	0.089
	(0.019)	(0.022)***	(0.027)**	(0.050)+
Girl	-0.130	-0.197	-0.166	-0.249
	(0.089)	(0.094)*	(0.098)+	(0.107)*
R2	0.12	0.11	0.12	0.08
N	1,178	1,117	1,171	1,090
		**Overnight stays**		
Active fathering	0.163	0.144	0.081	0.107
	(0.052)**	(0.049)**	(0.046)+	(0.047)*
Solo-fathering	0.149	0.063	0.070	0.062
	(0.051)**	(0.051)	(0.048)	(0.047)
Cohabiting	-0.113	-0.117	0.043	0.194
	(0.113)	(0.110)	(0.101)	(0.105)+
Child age	0.001	0.008	0.050	0.078
	(0.022)	(0.022)	(0.023)*	(0.041)+
Girl	-0.204	-0.128	-0.176	-0.286
	(0.096)*	(0.090)	(0.085)*	(0.089)**
R2	0.16	0.13	0.16	0.17
N	785	955	1,171	1,090

Source: Millennium Cohort Study. All models include additionally: child sex, father's age, qualifications, work status, health status, family income, London or not, mother's work status and health status, whether child has older or younger siblings, length of the parents' cohabiting / married relationship prior to the child's birth. + p<0.1; * p<0.05; ** p<0.01; *** p<0.001

Figure S1: Contact probability by time since separation among older and younger children

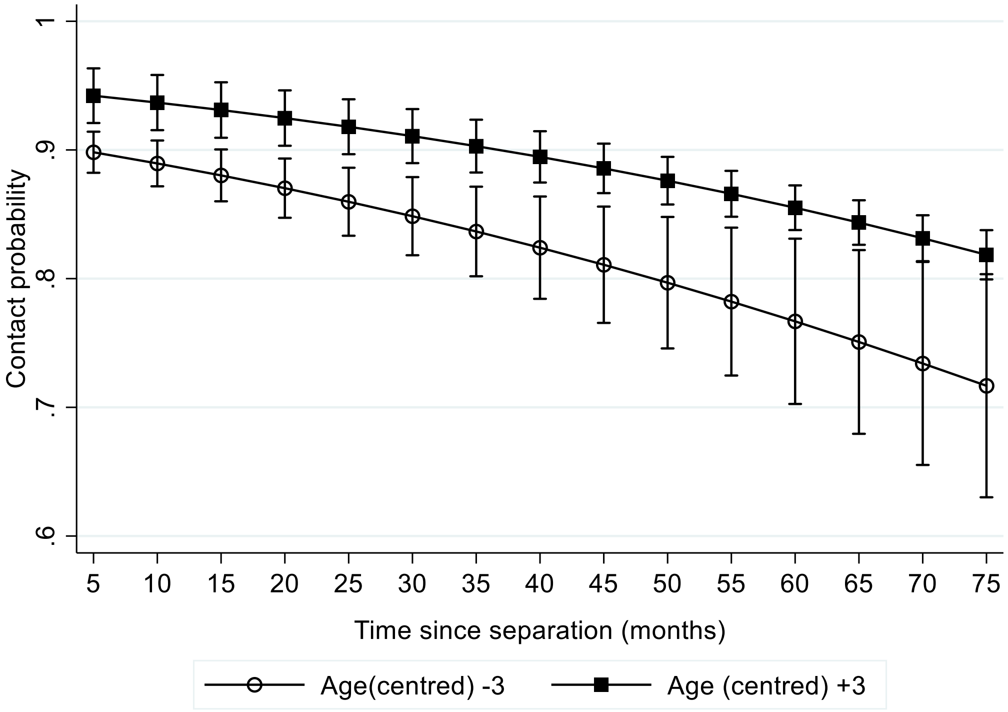

Note: the confidence intervals increase at larger times since separation for younger children since these become increasingly less plausible. For age differences, the left hand side of the chart provides a more relevant comparison.
